# Effects of bovine serum albumin on light activated antimicrobial surfaces†

**DOI:** 10.1039/c8ra04361b

**Published:** 2018-10-05

**Authors:** Cláudio Lourenço, Thomas J. Macdonald, Asterios Gavriilidis, Elaine Allan, Alexander J. MacRobert, Ivan P. Parkin

**Affiliations:** Materials Chemistry Research Centre, Department of Chemistry, University College London 20 Gordon St London WC1H 0AJ UK i.p.parkin@ucl.ac.uk; Department of Chemical Engineering, University College London Torrington Place London, WC1E 7JE UK; Division of Microbial Disease, UCL Eastman Dental Institute University College London 256 Gray's Inn Road London WC1X 8LD UK; Division of Surgery and Interventional Science, University College London, Royal Free Campus Rowland Hill Street London WC1E 6BT UK

## Abstract

Bovine serum albumin (BSA) is currently recommended as an interfering substance to emulate organic soiling, in evaluating the efficacy of disinfectants. The European Standard recommends 0.03% BSA to test clean conditions and 0.3% for dirty conditions. Reactive oxygen species are known to exert excellent antimicrobial activity with low specificity against a broad range of pathogens. Herein, we present our data from the first study of the effects of the addition of BSA on the antibacterial activity of light activated antimicrobial surfaces. Light activated antimicrobial surfaces were made from polyurethane swell-encapsulated with gold nanoparticles (AuNPs) coated with the light active triarylmethane dye, crystal violet (PU-AuNP-CV). The antibacterial efficacy of the antimicrobial substrates was tested against two strains of *Staphylococcus aureus* 8325-4, a well-characterised laboratory strain and MRSA 4742, a recent clinical isolate, in the presence of 0.1% to 1% BSA by irradiating the substrates with a fluorescent lamp (300 lux). After 6 hours of irradiation, the number of surviving bacteria was determined. The results showed that BSA reduced the antibacterial efficacy of all the PU-AuNP-CV surfaces with increasing BSA concentrations resulting in a progressive reduction in antibacterial activity towards the bacteria tested. However, the light activated surfaces did perform well at 0.1 and 0.25% BSA levels, showing they may have potential for real world environments with low levels of organic soiling.

## Abstract 2

In this work we demonstrate that our active surfaces still show antibacterial activity even with BSA at low light.

## Graphical abstract

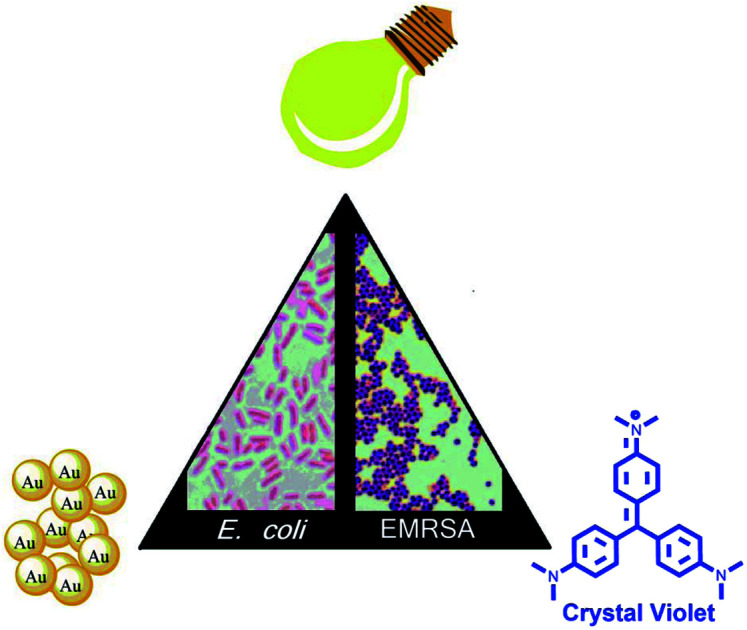

## Introduction

Hospital acquired infections (HAIs) present a considerable challenge due to the ability of pathogens to spread within healthcare environments and the increasing development of drug resistance.^1^ One of the most common ways in which bacteria are spread in hospital environments is through contact between surfaces, patients and healthcare workers. A key issue is that there are numerous touch surfaces within hospital environments which can harbour and spread microorganisms, including telephones, keyboards, furniture (bed rails and door handles), food trays, and implanted medical equipment such as catheters.^2–4^ Although established disinfection protocols may help prevent the build-up of bacteria on surfaces, some bacteria are able to resist disinfection and form potential reservoirs of infection.^5^ Methicillin-resistant *Staphylococcus aureus* (MRSA), methicillin-sensitive *Staphylococcus aureus* (MSSA), *Clostridium difficile* and *E.* coli are of major importance in healthcare associated infection. Their screening, monitoring and reporting are mandatory for healthcare facilities.^6^ One of the main concerns in the hospital environment is *Staphylococcus aureus* that is part of the microbiota of 30% of the population but can also be a dangerous pathogen that can cause bacteraemia and pneumonia among other infections. It has been reported that methicillin resistant strains of *S. aureus* can survive on surfaces for as long as 360 days. Apart from cleaning and disinfection there are not many effective ways adequate to deal with this organism.^7,8^*Escherichia coli* (*E. coli*) is a Gram-negative bacterium part of our gut microbiota. Gram-negative bacteria are of great concern due to the prevalence of antibiotic resistant strains.^9^*E. coli* alone is the major cause of bloodstream infections in England and responsible for the majority of community and hospital acquired infections. Among those are urinary tract infections and food poisoning.^10^

There are many reports of antimicrobial surfaces for the prevention or reduction of bacterial surface contamination.^3,11,12^ While most surfaces comprise either metal nanoparticles or light activated antibacterial components, a minority employ a combination of both. Our previous work has reported antibacterial surfaces made from polyurethane swell-encapsulated with gold nanoparticles (AuNPs), coated with a light active triarylmethane dye, crystal violet (CV).^13–15^ When thiol-capped 2 nm AuNPs are combined with photosensitised dyes (such as CV), a synergy of antibacterial efficacy is observed. We have previously shown that this enhancement in bactericidal activity is related to the size of the AuNPs, where 2 nm are the most effective at enhancing the antibacterial effect.^13^ Other studies using similar approaches using copper^14^ or zinc nanoparticles^15,16^ with light activated antimicrobial surfaces also demonstrate the potential of antibacterial surfaces for use in healthcare environments.

The antibacterial properties of light activated antimicrobial materials have been reported against representative Gram-positive and Gram-negative bacteria, and have shown on average 3 log (99.9% kill) and 1.5 (95% kill) log reduction in bacterial numbers, respectively.^15–18^ The activity of such materials can be fine-tuned by modifying the capping ligands in the AuNPs, the nanoparticle concentration and by the use of different dyes or combination of dyes.^13,16,17^ The antibacterial properties of such surfaces are activated by light, where during irradiation, immobilised dye molecules (CV) are promoted to an excited singlet state that can undergo inter-system crossing to a triplet state. The excited dye molecule can participate in a series of photochemical reactions that will lead to the generation of different reactive oxygen species (ROS).^19^ ROS is a term used to categorise an array of reactive molecules and free radicals that are generated by the incomplete reduction of oxygen. Such species are extremely reactive towards important biomolecular substrates such as amino acid residues, which can give rise to free radical oxidative chain reactions that are toxic to bacteria.^20–22^ In such systems, these photochemical reactions yield a range of different reactive oxygen species (ROS), including H_2_O_2_, *via* a type I or type II process.^14,15,18^

A study by Imlay and Linn^23^ with *E. coli* shows that at low concentrations of H_2_O_2_ (1–3 mM) bacteria were killed mostly by DNA damage. At higher concentrations the occurrence of more generalised damage was evident, and there was also evidence that the DNA damage was dependent on iron suggesting that, through the Fenton reaction, hydroxyl radicals or other ferrous products were taking part in the mechanism.^23,24^ Commonly, higher antibacterial activity is observed against Gram-positive bacteria compared to Gram-negative bacteria; the presence of a thicker cell wall and defence mechanisms such as ROS scavengers can increase the tolerance of organisms to the damage caused by ROS.^25,26^ One of the drawbacks of using ROS is their extreme reactivity towards organic compounds such as proteins, coupled with a short lifetime.^27,28^ In order to harness the full power of ROS, some manufacturers address this issue by relying on the use of ROS precursors and H_2_O_2_ activators to generate peracids and ROS *in situ* rather than including this in their formulation.^29–32^ Despite this, ROS are extremely effective in the removal of dirt, disinfection and, are currently employed in many situations including water treatment facilities, the paper industry, health facilities, and household cleaners among others.^27,29,33,34^

The European Standard EN 1276 “Chemical disinfectants and antiseptics – quantitative suspension test for the evaluation of bactericidal activity of chemical disinfectants and antiseptics used in food, industrial, domestic and institutional areas” recommends the testing conditions acceptable in Europe to determine the antibacterial activity of products released or to be released for the “general public”. Its scope mainly refers to liquid suspension testing with the use of pre-set conditions to allow comparison between data generated in different laboratories.^35^ The European Standard also considers the use of an interfering substance that will mimic the impurities/contamination present in the environment. For this purpose it recommends the use of bovine serum albumin (BSA) in order to mimic organic soiling.^36^ Serum albumin is the most abundant protein in the circulatory system of animals and humans possessing a wide range of functions such as, transport, free radical scavenging and osmotic pressure regulation^37,38^ and binding toxic substances.^39^ Furthermore, it is also recommended for the product to be tested under simulated clean (0.03% BSA) and dirty (0.3% BSA) conditions,^35^ thus making the test more robust and a better model of real case scenarios.

In this paper light activated antimicrobial surfaces were challenged with bacterial suspensions of a clinical isolate of methicillin-resistant *Staphylococcus aureus* (MRSA) and a methicillin sensitive strain of *S. aureus*, 8325-4 (ref. 40 and 41) (MSSA) as representative Gram-positive bacterium, and a clinical isolate of *E. coli* as a representative Gram-negative bacterium in the presence of various concentrations of BSA. The antibacterial efficacy of the surfaces was determined after irradiation by determining the number of surviving bacteria. We show that at low concentrations of BSA these surfaces work well (0–0.25% BSA), they function adequately at 0.5% BSA showing between 95–99% kill but are inactivated at 1% BSA and above. The tests were performed at low light intensities (300 lux) to simulate hospital ward conditions.^42^

## Materials and methods

### Materials chemistry characterisation

Transmission Electron Microscopy (TEM) was performed using a JEOL 2010 TEM operating at 200 kV. Image collection and processing was performed on a CCD with Gatan Digital Micrograph software. Particle size analysis was carried out using ImageJ software. X-ray photoelectron spectroscopy (XPS) was carried out using a Thermo Scientific K-alpha photoelectron spectrometer with monochromatic Al-K_α_ radiation to analyse the Au nanostructures in polyurethane. Peak positions were calibrated to carbon (285 eV) and plotted using the CasaXPS and qtiplot software.

### Synthesis of thiol-capped 2 nm AuNPs

AuNPs were synthesised according to a method previously described by Brust *et al.*^43^ Briefly, a 6 mL (30 mM) aqueous solution of chloroauric acid trihydrate (VWR, UK) was mixed with 16 mL (50 mM) tetraoctylammonium bromide (Sigma Aldrich, UK) in toluene. Once the gold salt was transferred to the organic phase, 40 μL (10.5 mM) of 1-dodecanethiol (Sigma Aldrich, UK) was added as a capping ligand. A solution of freshly prepared 5 mL (0.4 mM) sodium borohydride was added dropwise to the mixture, and the solution was left to stir for 3 hours. The organic layer was extracted and the AuNP solution was concentrated on a rotary evaporator (2 mL). 40 mL of ethanol (Sigma Aldrich, UK) was added to the concentrate and the AuNPs were stored for 4 hours at −18 °C, after which a black precipitate was formed. The AuNPs were centrifuged and concentrated to yield ∼1 mg mL^−1^ in toluene (Sigma Aldrich, UK) before further use.

### Polyurethane nanoparticle swell-encapsulation

The method used was adapted from the work of Noimark *et al.* (2015)^15^ and Sehmi *et al.* (2016)^17^ using the following: medical grade polyurethane (Branford, CT, USA), toluene analytical reagent grade (Fisher Chemical), crystal violet (CV) (Sigma-Aldrich).

First 1 × 1 cm polyurethane (PU) squares were immersed in a 1 mg mL^−1^ AuNP solution (synthesis described above) in toluene for 24 hours to swell-encapsulate. Then the squares were air dried on a clean glass surface for approximately 12 hours.

The polyurethane squares were then immersed in a 0.0001 M crystal violet solution for 72 hours, in the dark. The solution was gently stirred twice a day to circulate the dye between polymers and the samples were rinsed in deionised H_2_O and air dried in the dark for 24 hours.

A set of control polymers was also prepared using only the swell-encapsulation solvent (toluene).

### Antibacterial testing

The antibacterial testing was carried out as previously described^15,17^ with the addition of various concentrations of bovine serum albumin (BSA) (Sigma) to phosphate-buffered saline (PBS, Oxoid). Two strains of *S. aureus* were used: a clinical methicillin-resistant strain (MRSA 4742; obtained from P. Wilson, University College London Hospital), and *S. aureus* 8325-4 (ref. 40 and 41) and *E. coli* 1030, a multidrug resistant strain positive for both NDM and OXA-48-like carbapenemase genes (obtained from J. Wade, King's College Hospital, London). The strains were stored at −70 °C in BHI broth containing 20% (v/v) glycerol and propagated on mannitol salt agar (Oxoid) or MacConkey agar (Oxoid) for a maximum of two subcultures every two weeks.

The bactericidal properties of 1 × 1 cm PU squares (control and CV + AuNP) were tested against MRSA 4742, *S. aureus* 8325-4 and *E. coli* 1030. The inoculum was prepared by inoculating 10 mL BHI with a single colony and incubating for 18 hours (200 rpm, 37 °C). The bacteria were collected by centrifugation (20 °C, 4500*g*, 5 min) and washed twice using PBS and resuspended in 10 mL of PBS. The suspensions were then diluted 1000-fold into PBS with the desired concentration of BSA (5%, 2%, 1%, 0.5%, 0%) to attain an inoculum of ∼10^5^–10^6^ colony forming units (CFU) per mL. In each experiment the inoculum was confirmed by plating serial dilutions on agar in duplicate for viable counts. The test materials (PU + CV and PU + AuNP + CV) were inoculated with 25 μL of the inoculum containing ∼10^5^–10^6^ CFU mL^−1^, placed inside a Petri dish and irradiated using a conventional ceiling fluorescent lamp with an average intensity of 300 lux for a 6 hours period for the gram positives and 48 hours for the gram negatives. After irradiation, the inoculated samples were added to 450 μL PBS and vortexed to remove all the bacteria. Afterwards the resulting neat suspension and serial dilutions were plated on Columbia agar enriched with 5% horse blood and incubated for 24 hours (37 °C, 5% CO_2_) to determine the viable counts.

## Results and discussion

**Fig. 1:**
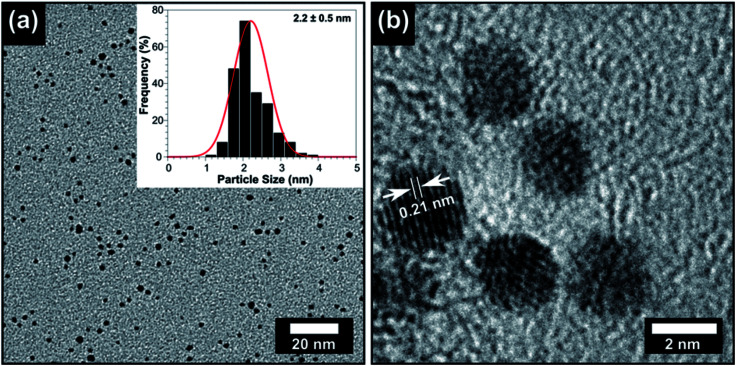
(a) TEM image of the AuNPs and the inset shows the particle size histogram. (b) high resolution-TEM of the AuNPs showing lattice spacing of 0.21 nm consistent with the {111} plane.

AuNPs were synthesised as described in the materials and Methods section,^13,43^ and their particle size was characterised using transmission electron microscopy (TEM). Fig. 1 shows the TEM and a particle size distribution of the AuNPs revealing their size of 2.2 ± 0.5 nm, which corresponds to previously reported methods.^13^ High-resolution TEM shows the AuNPs have a lattice spacing of 0.21 nm which is very close to the {111} plane.^44^ The polyurethane squares were swell-encapsulated using a 1 mg mL^−1^ AuNP solution in toluene for 24 hours and subsequently coated with CV in solution 0.001 M. After rinsing to remove excess CV, the polymer squares looked uniformly dark blue without any deposits or abnormal features on its surface. Prior to the antimicrobial testing, the polymers were carefully checked, visually, for any imperfections. The presence of AuNPs in polyurethane has been well characterised in our previous reports,^13^ however X-ray photoelectron spectroscopy (XPS) was also used in this work to confirm the presence of AuNPs in the polymer. In order to confirm that the AuNPs were not simply deposited on the surface of the polymer, a monatomic depth profile was measured. This involved using a calibrated ion beam that etched layers off the polymer, revealing subsurface information. The etching was performed for 200 seconds which corresponded to a penetration of ∼50 nm as per our previous report.^13^ Fig. 1S† verified the Au4f_(5/2)_ and Au4f_(7/2)_ peaks at 87.3 eV and 83.6 eV respectively. The AuNP peaks present in the Au4f spectra correspond to a spin orbital splitting of 3.7 eV. The Au4f_(7/2)_ peak at 83.6 eV is closely matched with that of metal Au^0^ which is typically present at 84.0 eV. The slight negative shift of 0.4 eV for Au^0^ can be due to the interaction of the organic capping ligands with the polyurethane surface.^45,46^

**Fig. 2:**
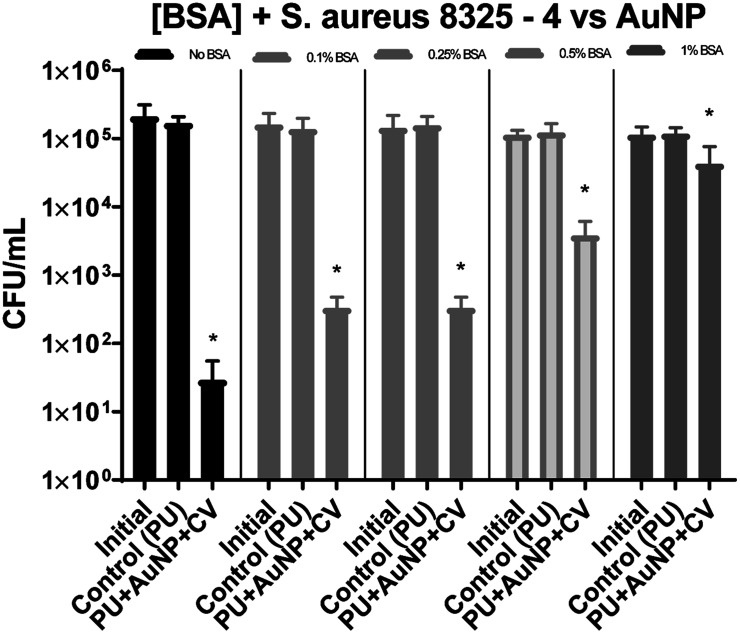
The numbers of *S. aureus* 8325-4 CFU recovered after irradiation at 300 lux, 6 hours. Initial – inoculum; control – untreated polyurethane (PU); PU + AuNP + CV – PU with encapsulated AuNP and coated with crystal violet. All experiments were carried out in duplicate and repeated three times. The data shows mean values + SD, statistical significance was determined using the Student's *t*-test comparing each test condition to its respective control (*p < 0.001).

For *S. aureus* 8325-4 (Fig. 2), in the absence of BSA, a 3.8 log reduction in bacterial numbers (99.9%) was obtained after 6 hours exposure to 300 lux irradiation. In the presence of either 0.1% or 0.25% BSA, the reduction in bacterial numbers was 2.1 log (99%) and only 1.5 log in the presence of 0.5% BSA. At 1% BSA, only a 0.5 log reduction in bacterial numbers was observed. This result shows that, as expected, by increasing the protein load within our test, we reduce the antibacterial efficacy of the material but importantly, some activity is retained under low soiling conditions.

**Fig. 3:**
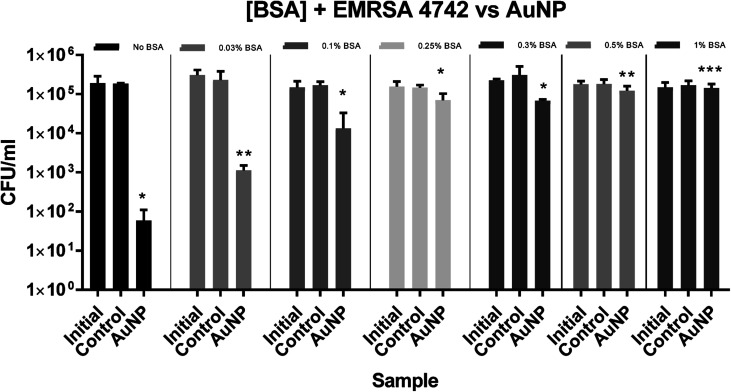
The numbers of EMRSA-4742 recovered after irradiation 300 lux, for 6 hours. Initial – inoculum; control – untreated polyurethane (PU); PU + AuNP + CV – PU with encapsulated AuNP and coated with crystal violet. All experiments were carried out in duplicate and repeated three times. The data shows mean values + SD, statistical significance was determined using the Student's *t*-test comparing each test condition to its respective control (*p < 0.005, **p = 0.001, ***p = 0.01).

For EMRSA4742, the inhibitory effect of BSA towards ROS-induced kill was more evident as shown in Fig. 3 and 5. In the absence of BSA, a log reduction of 3.7 in the number of bacteria was observed and a reduction in the bactericidal activity was again apparent in the presence of BSA. At a concentration of 0.1% BSA, PU + AuNP + CV showed a 1.5 log reduction in bacterial numbers and the bactericidal activity was gradually reduced with increasing BSA concentration: a 0.4 log reduction in bacterial numbers at 0.25% BSA compared to a 0.1 log reduction in the presence of 0.5% BSA.

**Fig. 4:**
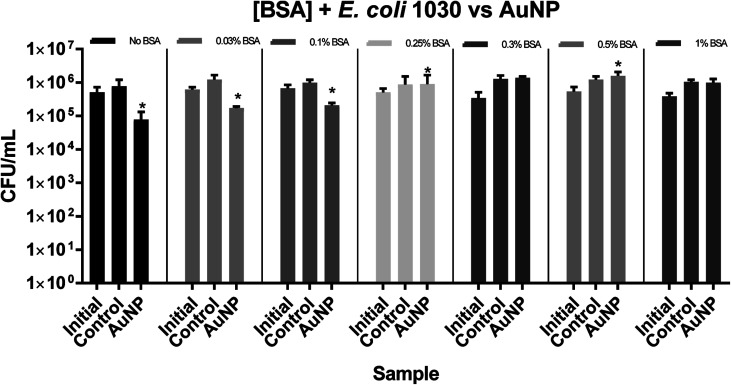
The numbers of *E. coli* 1030 recovered after irradiation 300 lux, for 48 hours. Initial – inoculum; control – untreated polyurethane (PU); PU + AuNP + CV – PU with encapsulated AuNP and coated with crystal violet. All experiments were carried out in duplicate and repeated three times. The data shows mean values + SD, statistical significance was determined using the Student's *t*-test comparing each test condition to its respective control (*p < 0.05).

**Fig. 5:**
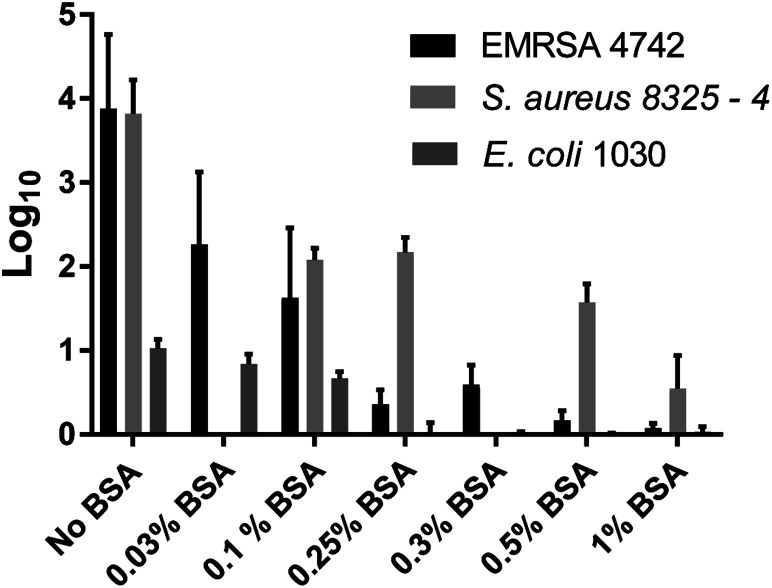
log_10_ reduction of EMRSA 4742, *S. aureus* 8325-4 and *E. coli* 1030 observed after irradiation 300 lux, 6 hours for *S. aureus* 8325-4 and MRSA 4742. With the addition of BSA these reductions are decreased differently between each tested organism. The data in the graph shows mean values + the bars show the SD.

For *E. coli* 1030, a 1 log reduction in bacterial numbers was observed in the absence of BSA and a statistically significant reduction in bacterial numbers was only apparent at concentrations of 0.1% BSA and below, as shown in Fig. 4 and 5.

Serum albumin can bind to a wide range of molecules and some of its antioxidant properties arise from the binding and inactivation of oxidative molecules.^39,47^

It is interesting that in the absence of BSA, the two strains of *S. aureus* showed similar susceptibility to the nanoparticles whereas in the presence of 0.25% BSA and above, the clinical EMRSA strain was significantly more resistant. The reason for this difference requires further investigation.

Previous studies^13^ using thiol capped AuNP encapsulated in polyurethane and also coated in CV showed excellent antibacterial activity towards *S. aureus* and *E. coli* demonstrating an approximately 6 log reduction in the bacterial numbers for both organisms within 2 hours for *S. aureus* and 6 hours for *E. coli*. This higher antimicrobial activity can be attributed to the fact that 6000 lux were used as irradiation energy.

An important and novel aspect of the work reported here is that we used 300 lux which is equivalent to ambient light in a hospital ward and 20 times less intense than our previous reports.^4,13,14,16^ Obviously the antimicrobial properties of the surfaces would be more effective at higher light intensities.^13^ For example, normal day-light ranges between 1000–10 000 lux. Therefore, the antimicrobial efficacy of such materials will be increased under natural light conditions.

## Conclusions

Polyurethane swell encapsulated with gold nanoparticles coated with crystal violet (PU-AuNP-CV) substrates showed a significant level of light-induced antibacterial activity against the tested organisms. Under a low visible light intensity of 300 lux, the substrates showed log reductions of 3.7 and 3.8 for EMRSA-4742 and *S. aureus* 8325-4 respectively that correlates with the elimination of 99.99% of the inoculated bacteria after exposure to 300 lux light intensity for 6 hours. In the case of *E. coli* 1030 only 1 log reduction was observed after 48 hours irradiation with 300 lux. It is well known that Gram-negative bacteria are more resilient to ROS presumably as a result of reduced penetration across the double membrane comprising its cell wall. Light alone in the absence of CV and/or AuNPs induced negligible kill.

Furthermore, we conducted a systematic study of the effect of adding BSA at different concentrations and demonstrated that its addition impaired the antibacterial efficacy of our material which is attributed to the antioxidant properties of BSA which scavenges the ROS released at the polymer surface. Importantly, the data presented shows that these materials retain some activity even in the presence of low soiling and at low light levels.

In conclusion, we find that light-activated antimicrobial surfaces are effective and show statistically significant bactericidal activity at low levels of light illumination (300 lux) under both European Standard clean (0.03% BSA > 99% kill) and dirty (0.3% BSA > 99% kill) conditions, against the Gram-positive bacteria tested. Under extremely soiled conditions, three-fold higher than the European Standard recommendation, they are still functional, retaining approximately 50% activity.

## Conflicts of interest

There are no conflicts of interest to declare.

## Abbreviations

BSA: Bovine Serum Albumin
ROS: Reactive Oxygen Species
PU-AuNP-CV: Polyurethane swell-encapsulated with gold nanoparticles coated with crystal violet
EMRSA: Epidemic Methicillin Resistant *Staphylococcus aureus*

## Supplementary Material

RA-008-C8RA04361B-s001
