# Supplementary material for: Effects of bovine serum albumin on light activated antimicrobial surfaces
Source: RSC Adv. 2018 Oct 5;8(60):34252–8. doi: 10.1039/c8ra04361b (PMC9087004; doi:10.1039/c8ra04361b)
Supplement: RA-008-C8RA04361B-s001 [file RA-008-C8RA04361B-s001.pdf]

Electronic Supplementary Material (ESI) for RSC Advances.
This journal is © The Royal Society of Chemistry 2018

Supporting Material

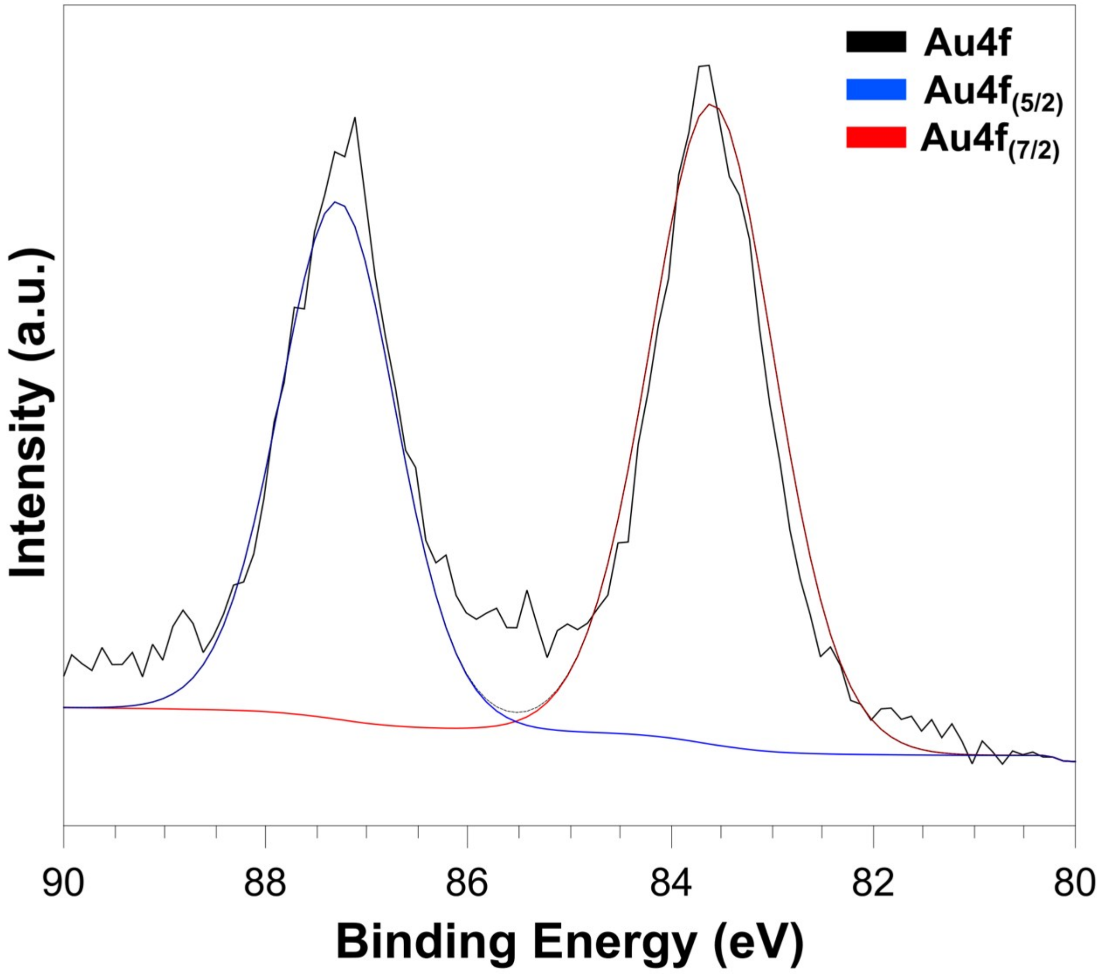

Figure 1S: XPS of AuNPs after swell encapsulation into polyurethane. The Au4f peaks confirm the presence of AuNPs into the polymer.

Antimicrobial Testing Methods Illustration

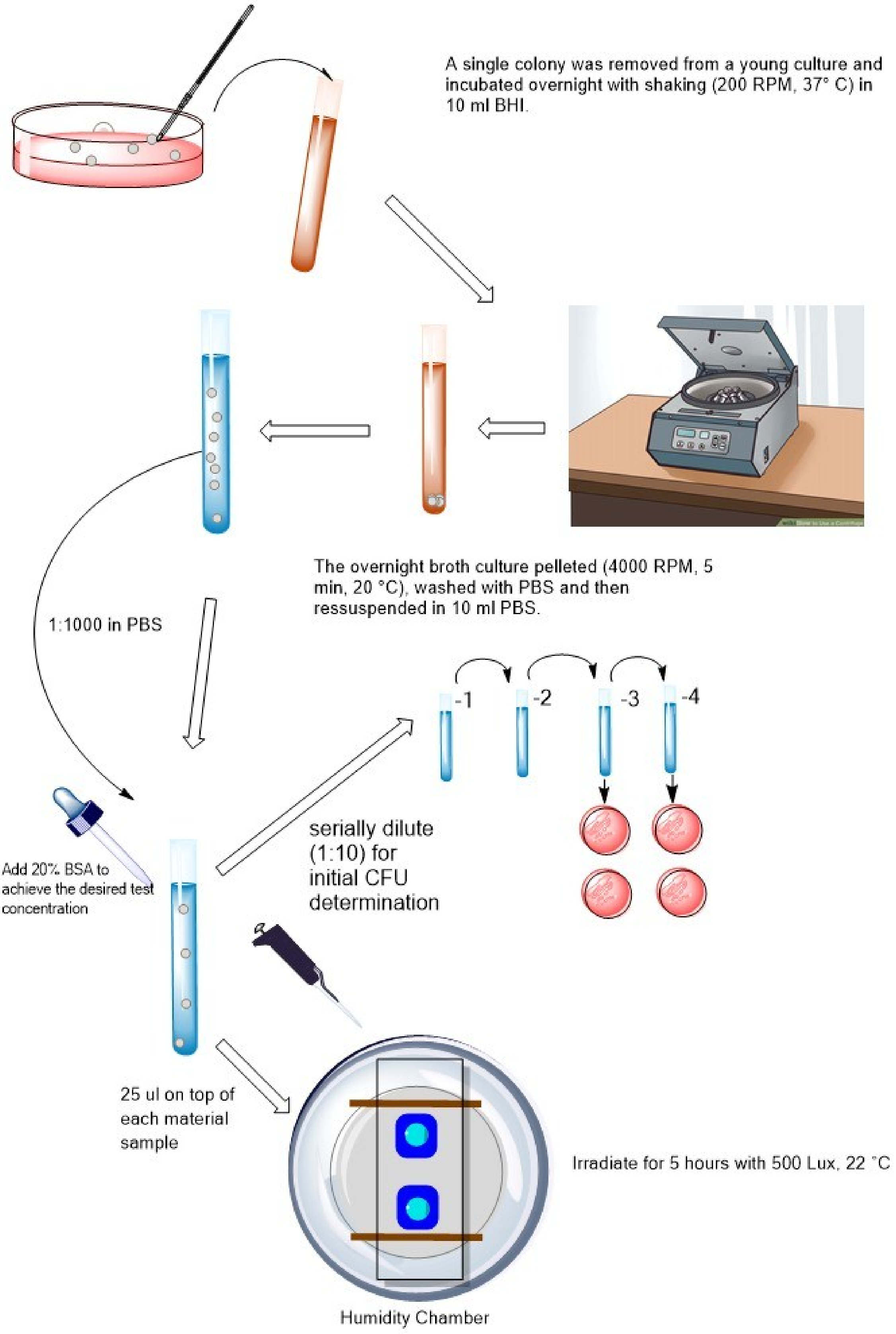

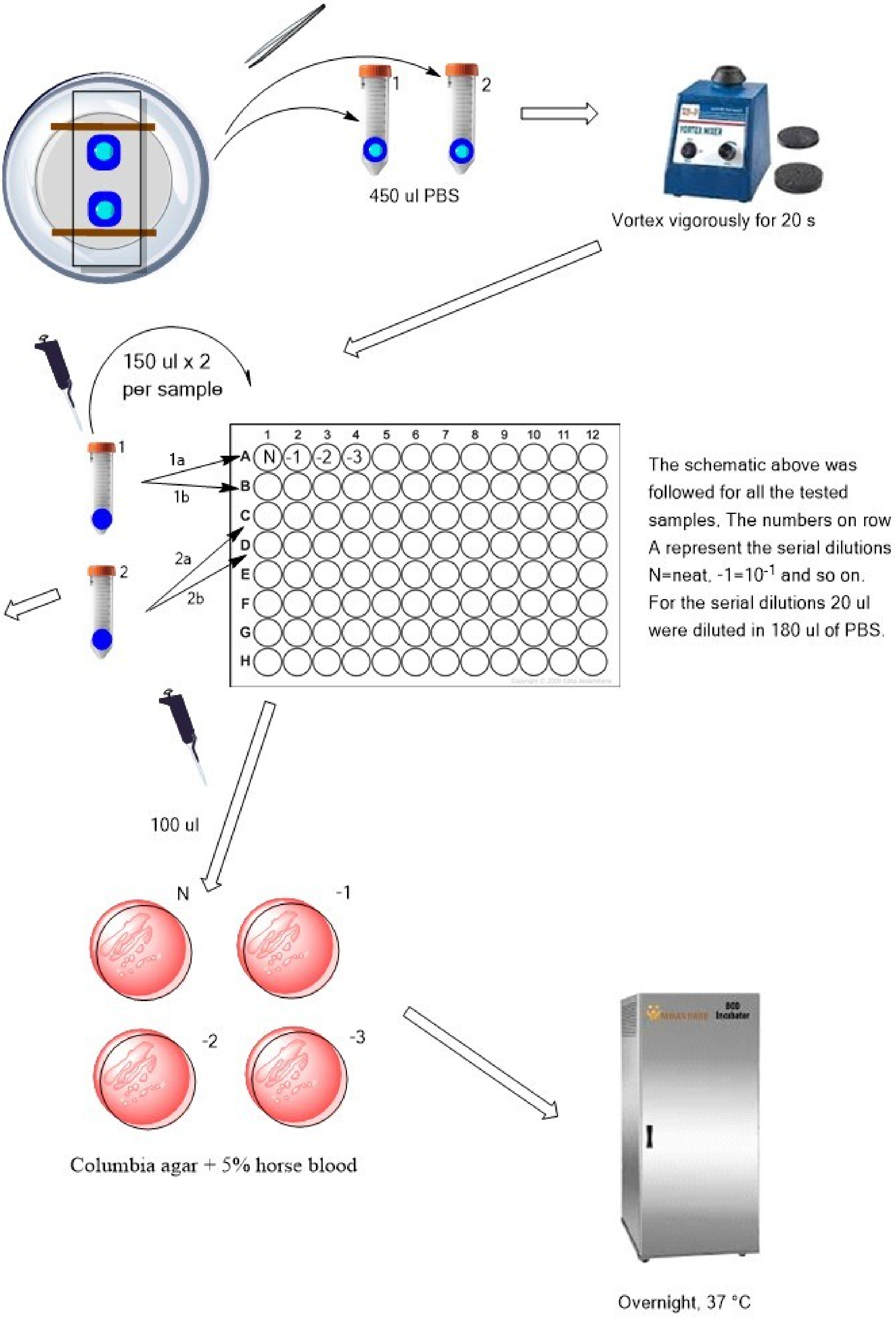

1
2
450 ul PBS
Vortex vigorously for 20 s
150 ul x 2
per sample
1
2
1a
1b
2a
2b
1 2 3 4 5 6 7 8 9 10 11 12
A B C D E F G H
N -1 -2 -3
The schematic above was followed for all the tested samples. The numbers on row A represent the serial dilutions N=neat, -1=10^{-1} and so on. For the serial dilutions 20 ul were diluted in 180 ul of PBS.
100 ul
N
-1
-2
-3
Columbia agar + 5% horse blood
Overnight, 37 °C
